# 伴有遗传易感的髓系肿瘤：Shwachman-Diamond综合征并骨髓增生异常综合征二例

**DOI:** 10.3760/cma.j.issn.0253-2727.2021.06.011

**Published:** 2021-06

**Authors:** 建平 李, 馨 赵, 康 周, 蕾 叶, 洋 李, 园 李, 广新 彭, 丽萍 井, 莉 张, 凤奎 张

**Affiliations:** 中国医学科学院血液病医院（中国医学科学院血液学研究所），实验血液学国家重点实验室，国家血液系统疾病临床医学研究中心，天津 300020 State Key Laboratory of Experimental Hematology, National Clinical Research Center for Blood Diseases, Institute of Hematology & Blood Diseases Hospital, Chinese Academy of Medical Sciences & Peking Union Medical College, Tianjin 300020, China

Shwachman-Diamond综合征（SDS）由Shwachman和Diamond在1964年首先报道[1]，临床表现为胰腺外分泌不足、血液系统异常及骨病等。胰腺功能障碍通常在婴幼儿期以脂肪泻起病，随年龄增长可逐渐缓解，至青少年时期基本消失。血液系统主要表现为不同程度的三系血细胞减少和骨髓增生异常综合征（MDS）/急性髓系白血病（AML）的遗传易感性。临床报道多以儿童病例为主，以MDS/AML就诊而确诊的病例报道少见。现将我院伴有发育异常的2例年轻MDS且最终诊断为SDS的患者报道如下，并复习相关文献，提高对本病认识。

## 病例资料

例1，女，22岁，入院前4个月常规体检发现血细胞减少，血常规示：WBC 2.63×10^9^/L，HGB 86 g/L，PLT 273×10^9^/L，骨髓穿刺涂片示增生活跃，原始粒细胞占0.080，粒、红、巨核三系增生伴形态发育异常；染色体荧光原位杂交（FISH）示存在克隆性5q−、7q−、+8异常。外院诊断为MDS-难治性贫血伴原始细胞增多（RAEB）Ⅰ型（IPSS高危）。应用地西他滨20 mg·m^−2^·d^−1^×5 d治疗1个疗程，患者出现重度粒细胞缺乏，反复发热，体温达39 °C，抗感染治疗效果不佳。PET-CT示：①肝脏、脾脏弥漫性肿大并多发低密度灶；②胰腺萎缩并脂肪变。复查骨髓示增生活跃，原始粒细胞占0.165，诊断为MDS-RAEBⅡ型，后患者就诊于我院。患者自幼间断腹泻，身材发育较同龄人延迟。父母健在，父亲、叔叔身材较矮小（身高150～160 cm），血常规不详，其母身高发育正常。患者祖父母均身体健康，身高发育正常。体格检查：身材矮小（身高150 cm，体重37.5 kg），重度贫血貌，周身皮肤无异常色素沉着，双下肢轻度水肿。血常规：WBC 1.99×10^9^/L，ANC 0.24×10^9^/L，HGB 56 g/L，PLT 66×10^9^/L，网织红细胞比例（Ret）0.39％。骨髓象：增生活跃，原始粒细胞占0.225，可见粒、红系形态异常。染色体核型：43-46,XX,−4,−5,−7,−9,−12,+2−5mar,inc［cp8］。FISH检查示5q−（92％）、7q−（80％）。骨髓流式细胞术免疫表型：异常细胞占有核细胞23.3％，表达CD34、CD117、CD13、CD33；弱表达CD38、CD7；不表达CD19、CD4、CD14、CD56；为异常髓系原始细胞，诊断：MDS转AML。MDS相关基因NPM1、DNMT3A-ZNF、DNMT3A-MTase、SRSF2、SF3B1、U2AF1、IDH1-EXON4、IDH-EXON4均阴性。彗星试验：彗星细胞率15％，未见凋亡细胞。二代测序：患者SBDS基因c.258+2T>C纯合突变。行患者亲代验证：父亲SBDS基因c.258+2T>C纯合突变；母亲SBDS基因c.258+2T>C杂合突变。诊断：①SDS；②AMLM_2_（MDS转化）；③肝脾念珠菌病。患者入院后持续粒细胞缺乏［（0.1～0.3）×10^9^/L］、高热，抗感染治疗无效，血常规无恢复。2个月后死于感染。

例2，女，13岁，入院前2个月因发热就诊于当地医院，血常规示：WBC 1.0×10^9^/L，HGB 118 g/L，PLT 70×10^9^/L。经抗感染治疗，患者发热消退，复查血常规仍为全血细胞减少，入住我院。患者幼时慢性腹泻，父亲42岁死于肝癌，母亲及姐姐体健。体格检查：身材矮小（身高142 cm，体重32 kg），贫血貌，心肺正常，肝脾不大。腹部B超：胰腺实质回声增强，提示胰腺脂肪化。血常规：WBC 2.81×10^9^/L，HGB 122 g/L，PLT 67×10^9^/L。骨髓象：增生明显活跃，粒系占0.565，核左移，可见颗粒增多增粗；红系占0.240，可见巨幼样变、花瓣核幼红细胞；巨核细胞207个，可见单圆核及多圆核巨核细胞，血小板少见。骨髓染色体核型：46,XX,del（20）（q11）[20]；骨髓免疫分型：髓系原始细胞0.14％，部分中晚幼粒细胞及单核细胞表达CD56；红系CD71/CD36表达减弱。外周血染色体体质性异常检测：46,XX,del（20）（q11.2q13.3）[3]/46,XX[17]。患者外周血淋巴细胞彗星细胞率16％。二代测序：患者及其姐姐均为SBDS基因c.258+2T>C杂合突变，母亲SBDS基因无异常。结合患者身材矮小、幼时腹泻、胰腺脂肪化、MDS发病年龄轻及基因检测结果，诊断：①SDS；②MDS伴多系血细胞发育异常（MDS-MLD）。患者未进行特殊治疗，定期监测血常规无下降趋势，病情平稳。

## 讨论及文献复习

SDS是一种罕见的常染色体隐性遗传病，90％以上患者可检测到SBDS基因突变，该基因位于染色体7q11，最常见的突变形式为c.183-184TA>CT和c.258+2T>C[2]。近两年有文献报道DNAJC21、EFL1和SRP54基因突变也可出现类似SDS临床表现[3]。SBDS基因编码蛋白影响核糖体的形成和细胞有丝分裂中纺锤体的稳定性[4]，并不表现染色体断裂增加[5]。本文中2例MDS患者均为青少年，伴有身材矮小、胰腺脂肪化，血液系统表现为MDS/AML及SBDS基因突变。其中，例1 SBDS基因经典位点纯合突变，诊断明确；例2 SBDS基因为杂合突变，结合相应临床表现可诊断本病。其基因检测结果可能与二代测序方法固有检测缺陷有关，不能检测大片段缺失或内含子剪切位点突变[6]。2例患者突变类型均为c.258+2T>C突变，与文献报告一致，并且彗星试验均无显著异常，支持SDS患者染色体断裂无明显增加。

SDS本身存在遗传不稳定性，造血细胞易发生异常克隆，而异常克隆最终导致MDS/AML转化，中位转化年龄在19～20岁[7]。Lindsley等[8]对国际血液与骨髓移植研究组入组的1514例成人MDS患者进行靶向突变分析，发现小于40岁的患者SBDS基因突变率显著高于40岁以上患者（2％对<1％，*P*<0.001）；虽然7例存在SBDS双等位基因突变的患者中仅2例临床诊断为SDS，但多个证据支持这些患者的SBDS突变为胚系突变；相对于SBDS单等位基因突变或无SBDS突变者，存在SBDS双等位基因突变的MDS患者均非常年轻［中位年龄25.1（18.2～38.2）岁］、身高显著减低。可见在年轻成人MDS患者中SDS的漏诊率仍非常高（5/7）。我们报告2例最终诊断为SDS的年轻MDS患者，病史中均出现不同程度延误诊断，提示对于儿童或年轻MDS/AML患者，特别是身材矮小或发育异常的患者，应警惕SDS可能，适时进行二代测序检测有助于明确诊断。同时，有研究发现SDS常合并TP53、IDH1基因突变，可能促进其MDS/AML转化，同时提示合并不同的基因突变可能与不同的预后相关[8]–[10]。

SDS合并血液系统恶性疾病转化者预后差，针对MDS/AML的化疗疗效不确切，且治疗相关不良反应较重[11]。例1在诊断MDS-RAEB后予化疗，长时间粒细胞缺乏，合并严重感染而死亡。异基因造血干细胞移植治疗为目前唯一可能治愈SDS血液系统并发症的治疗方案，但总体疗效不佳。Bhatla等[12]采用减低强度预处理方案联合同胞或无关供者异基因造血干细胞移植治疗可提高患者存活率。虽然体细胞突变分析（包括TP53分析）现可在临床上使用，但还不清楚如何利用这些信息指导治疗计划，仍需进行克隆进化与临床结果的纵向研究[11]。

综上所述，SDS患者在早期即可能出现血细胞减少和较典型胰腺功能障碍表现（如脂肪泻），多在婴儿期确诊。然而，胰腺功能可随着年龄增长逐渐改善，青少年时期基本消失，临床上以血细胞减少就诊而诊断为MDS/AML，易出现SDS延误诊断或漏诊。SDS相关年轻MDS患者常规治疗预后差，需要及时做出诊断，提高对本病认识至关重要。

## References

[1] Shwachman H, Diamod LK, Oski FA (1964). The Syndrome of pancreatic insufficiency and bone marow dysfunction[J]. J Pediatr.

[2] Boocock GR, Morrison JA, Popovic M (2003). Mutations in SBDS are associated with Shwachman-Diamond syndrome[J]. Nat Genet.

[3] Carapito R, Konantz M, Paillard C (2017). Mutations in signal recognition particle SRP54 cause syndromic neutropenia with Shwachman-Diamond-like features[J]. J Clin Invest.

[4] Huang JN, Shimamura A (2011). Clinical spectrum and molecular pathophysiology of Shwachman-Diamond syndrome[J]. Curr Opin Hematol.

[5] Maserati E, Minelli A, Pressato B (2006). Shwachman syndrome as mutator phenotype responsible for myeloid dysplasia/neoplasia through karyotype instability and chromosomes 7 and 20 anomalies[J]. Genes Chromosomes Cancer.

[6] 刘 嵘, 师 晓东, 刘 子勤 (2010). Shwachman-Diamond综合征一例临床和基因分析[J]. 中华血液学杂志.

[7] Donadieu J, Leblanc T, Bader Meunier B (2005). Analysis of risk factors for myelodysplasias, leukemias and death from infection among patients with congenital neutropenia. Experience of the French Severe Chronic Neutropenia Study Group[J]. Haematologica.

[8] Lindsley RC, Saber W, Mar BG (2017). Prognostic Mutations in Myelodysplastic Syndrome after Stem-Cell Transplantation[J]. N Engl J Med.

[9] Xia J, Miller CA, Baty J (2018). Somatic mutations and clonal hematopoiesis in congenital neutropenia[J]. Blood.

[10] Mourad S, Bilodeau M, Roussy M (2019). IDH1 as a Cooperating Mutation in AML Arising in the Context of Shwachman-Diamond Syndrome[J]. Front Oncol.

[11] Myers KC, Furutani E, Weller E (2020). Clinical features and outcomes of patients with Shwachman-Diamond syndrome and myelodysplastic syndrome or acute myeloid leukaemia: a multicentre, retrospective, cohort study[J]. Lancet Haematol.

[12] Bhatla D, Davies SM, Shenoy S (2008). Reduced-intensity conditioning is effective and safe for transplantation of patients with Shwachman-Diamond syndrome[J]. Bone Marrow Transplant.

